# Acute health-related quality of life outcomes and systemic inflammatory markers following contemporary breast cancer surgery

**DOI:** 10.1038/s41523-022-00456-4

**Published:** 2022-08-08

**Authors:** Arielle S. Radin, Julienne E. Bower, Michael R. Irwin, Arash Asher, Sara A. Hurvitz, Steve W. Cole, Catherine M. Crespi, Patricia A. Ganz

**Affiliations:** 1 Department of Psychology, University of California, Los Angeles (UCLA), Los Angeles, CA USA; 2 Jonsson Comprehensive Cancer Center, UCLA, Los Angeles, CA USA; 3 Department of Psychiatry and Biobehavioral Sciences, UCLA, Los Angeles, CA USA; 4 Cousins Center for Psychoneuroimmunology, Semel Institute for Neuroscience and Human Behavior, UCLA, Los Angeles, CA USA; 5 Departments of Medicine and Physical Medicine and Rehabilitation, Cedars Sinai, Los Angeles, CA USA; 6 Department of Medicine, David Geffen School of Medicine at UCLA, Los Angeles, CA USA; 7 Department of Biostatistics, UCLA-Fielding School of Public Health, Los Angeles, CA USA; 8 Department of Health Policy & Management, UCLA-Fielding School of Public Health, Los Angeles, CA USA

**Keywords:** Surgical oncology, Breast cancer

## Abstract

Contemporary breast cancer surgical procedures vary greatly by the amount of tissue removed, anesthesia time, and reconstruction. Despite historical literature comparing the health-related quality of life (HRQOL) after lumpectomy and mastectomy, HRQOL data are limited regarding contemporary surgical procedures. Further, biological processes (e.g., inflammation) associated with HRQOL outcomes have not been described. We conducted two studies to examine differences in post-operative physical and mental functioning, pain, fatigue, and systemic inflammatory markers including interleukin (IL)-6, tumor necrosis factor (TNF)-α, and C-reactive protein (CRP) in women with early-stage breast cancer. Study 1 assessed women before and after surgery (*n* = 27) and Study 2 used a large cross-sectional sample (*n* = 240) to confirm findings from Study 1 and included a no-surgery comparison group. In Study 1, women who received mastectomy had lower physical functioning than lumpectomy (*ps* < 0.05), and those who received bilateral mastectomy had worse pain (*p* < 0.01) and fatigue (*p* = 0.029) than lumpectomy. Results were replicated in Study 2: mastectomy groups exhibited poorer physical functioning (*ps* < 0.01) and greater pain (*ps* < 0.001) than lumpectomy, and bilateral mastectomy was associated with worse fatigue (*p* < 0.05). Women who received bilateral mastectomy had higher levels of CRP than lumpectomy (*p* < 0.01) and higher TNF-α than the no-surgery group (*p* < 0.05). All surgery groups exhibited higher IL-6 than no-surgery (*ps* < 0.05). More extensive surgery is associated with poorer postoperative HRQOL. As compared to lumpectomy and no-surgery, mastectomy is associated with higher concentrations of systemic inflammatory markers.

## Introduction

The surgical management of breast cancer has dramatically changed over the past three decades. For more than a century, mastectomy (modified/simple or Halsted radical, both with complete axillary dissection) was the surgery of choice; however, driven by the pioneering work of Dr. Bernard Fisher and the earlier detection of breast cancer, less extensive surgical procedures have replaced these more extensive procedures for many patients^1,2^. Contemporary breast cancer surgery varies widely; for example, from small surgical excisions of tumors that are <1 cm (lumpectomy) with or without sentinel node biopsy, to larger segmental mastectomy with or without axillary dissection, to mastectomy with or without axillary dissection, to skin sparing mastectomy with an implant, to skin sparing mastectomy with autologous reconstruction, to bilateral mastectomy with or without reconstruction. This range in the extent of surgery leads to highly variable amounts of tissue manipulation and injury during surgery, as well as wide ranges in the duration of anesthesia. Breast-conserving surgery is as efficacious as mastectomy for disease-free survival for early-stage disease^1–5^, with mastectomy only being advised when the patient has a medical condition that precludes radiation therapy, has a hereditary germline mutation that warrants preventive risk reduction surgery, has inflammatory cancer, multifocal/multicentric breast cancers in which breast conservation is not feasible, or a very large tumor where the cosmetic result of limited surgery would not be acceptable. Currently, other factors frequently influence the choice of more extensive surgery; these include the patient’s fear of recurrence, perceived survival benefit, media influence, desire to reduce the need for surveillance imaging, and cosmetic considerations^6,7^. The rise in the use of more extensive surgery has been influenced by the availability of newer breast reconstruction techniques, as well as the more widespread use of immediate reconstruction at the time of mastectomy.

Historic studies in the 1980s and 1990s compared health-related quality of life (HRQOL) in women treated with lumpectomy vs mastectomy^8,9^, and found few differences between these two surgical approaches, except for body image and sexual functioning^10–13^. There is little information available for clinicians and patients today on the acute effects of the varied contemporary breast cancer surgical treatments^14–16^ and whether or not there are treatment-related differences in post-operative physical and mental functioning and symptoms (e.g., increased fatigue and pain). Additionally, biological processes that might drive surgical treatment-related differences in HRQOL are currently unknown. Some investigations suggest that systemic inflammation increases following breast cancer surgery^17^ which may underlie decreased functioning and increased symptoms^18–20^; however, there is no information about how increases in systemic inflammation differ across different surgical procedures.

The degree to which the extent of primary surgery (lumpectomy, unilateral or bilateral mastectomy) is associated with post-treatment HRQOL and systemic inflammatory markers is the focus of the studies reported here. We examine these questions by presenting data from two separate studies: one that assessed women ~1 week before and 2 weeks after surgery in a small sample, and a second that provides confirmation of the findings noted in the first study using cross-sectional data from a larger sample of women assessed approximately one month following surgery.

## Results

### Study 1

#### Patients

Characteristics of participants for both studies are shown in Table 1. A total of 27 women enrolled in Study 1 and completed the pre- and post-surgery questionnaires. Of these women, 23 had blood available for immune assays at both the pre- and post-surgery visits. The pre-surgery visit took place an average of 8 days before surgery and the post-surgery visit took place an average of 13 days following surgery. All women receiving mastectomy (*n* = 9) had immediate reconstruction (*n* = 5, deep inferior epigastric perforators or superficial epigastric artery flap; *n* = 4, implant-based). Means and standard deviations for HRQOL and inflammatory outcomes based on reconstruction type are presented in Supplementary Table 3. Women who received bilateral mastectomies were on average younger than both those receiving lumpectomy and unilateral mastectomy, but these differences were not statistically significant based on ANOVA (*p*s > 0.05).

**Table 1:** Demographic and clinical characteristics.

	Study 1 (surgical symptoms study)				Study 2 (RISE study)				
	Lumpectomy (*n* = 18, 66.7%)	Unilateral mastectomy (*n* = 4, 14.8%)	Bilateral mastectomy (*n* = 5, 18.5%)	Total (*n* = 27)	No surgery (*n* = 25, 10.4%)	Lumpectomy (*n* = 155, 64.6%)	Unilateral mastectomy (*n* = 19, 7.9%)	Bilateral mastectomy (*n* = 41, 17.1%)	Total (*n* = 240)
Age, years (mean, SD)	56.2 (11.4)	52.8 (9.8)	44.6 (7.5)	53.6 (11.2)	48.8 (10.8)	58.1 (11.3)	57.4 (9.4)	51.6 (10.1)	55.93 (11.34)
Race/ethnicity (*n*, %)									
Non-Hispanic White	15 (83.3%)	3 (75%)	5 (100%)	23 (85.2%)	16 (64%)	113 (72.9%)	12 (63.2%)	29 (70.7%)	170 (70.83%)
Non-Hispanic Black	1 (5.6%)	0 (0%)	0 (0%)	1 (3.7%)	0 (0%)	8 (5.2%)	1 (5.3%)	2 (4.9%)	11 (4.58%)
Non-Hispanic Asian	1 (5.6%)	1 (25%)	0 (0%)	2 (7.4%)	4 (16%)	15 (9.7%)	3 (15.8%)	7 (17.1%)	29 (12.08%)
Non-Hispanic Other	1 (5.6%)	0 (0%)	0 (0%)	1 (3.7%)	2 (8%)	3 (1.9%)	1 (5.3%)	1 (2.4%)	7 (2.92%)
Hispanic	0 (0%)	0 (0%)	0 (0%)	0 (0%)	3 (12%)	16 (10.3%)	2 (10.5%)	2 (4.9%)	23 (9.58%)
Income, over 100,000 (*n*, %)	12 (66.7%)	2 (50%)	2 (40%)	16 (59.3%)	16 (76%)	82 (52.9%)	9 (47.4%)	22 (53.7%)	129 (53.75%)
BMI (mean, SD)	27.3 (6.4)	21.5 (0.6)	25.2 (2.3)	26.1 (5.7)	25.5 (4.8)	26.0 (6.0)	25.1 (6.7)	23.6 (4.5)	25.5 (5.8)
Education (*n*, %)									
High School	1 (5.6%)	0 (0%)	1 (20%)	2 (7.41%)	3 (12%)	46 (29.7%)	4 (21.1%)	12 (29.3%)	65 (27.08%)
Associates or college	10 (55.6%)	1 (25%)	2 (40%)	13 (48.2%)	16 (76%)	60 (38.7%)	7 (36.8%)	18 (43.9%)	101 (42.08%)
Post-graduate	7 (38.9%)	3 (75%)	2 (40%)	12 (44.4%)	6 (24%)	49 (31.6%)	8 (42.1%)	11 (26.8%)	74 (30.83%)
Employed (*n*, %)	10 (55.6%)	4 (100%)	4 (80%)	18 (66.7%)	20 (80%)	89 (57.4%)	9 (47.4%)	27 (65.9%)	145 (60.42%)
Stage (*n*, %)									
0	4 (22.2%)	0 (0%)	2 (40%)	6 (22.2%)	N/C	22 (14.2%)	0 (0%)	4 (9.8%)	26 (10.83%)
I	5 (27.8%)	1 (25%)	2 (40%)	8 (29.6%)	N/C	82 (52.9%)	10 (52.6%)	22 (53.7%)	114 (47.50%)
II	8 (44.4%)	3 (75%)	1 (20%)	12 (54.5%)	N/C	38 (24.5%)	7 (36.8%)	11 (26.8%)	56 (23.33%)
IIIA	1 (5.6%)	0 (0%)	0 (0%)	1 (3.7%)	N/C	5 (3.2%)	1 (5.3%)	3 (7.3%)	9 (3.75%)
Reconstruction type									
Autologous	N/A	2 (50%)	3 (60%)	5 (55%)	N/A	N/C	N/C	N/C	N/C
Implant-based	N/A	2 (50%)	2 (40%)	4 (45%)	N/A	N/C	N/C	N/C	N/C
Nodal surgery type									
Sentinel lymph node biopsy only	13 (72.2%)	2 (50%)	5 (100%)	20 (74.1%)	N/A	107 (69.0%)	11 (57.9%)	17 (41.5%)	135 (56.3%)
Axillary dissection	1 (5.6%)	1 (25%)	0 (0%)	2 (7.4%)	N/A	31 (20.0%)	4 (21.1%)	18 (43.9%)	53 (22.1%)
Anti-inflammatory Medications	Pre-surgery: 1 (5.6%) Post-surgery: 1 (5.6%)	Pre-surgery: 0 (0%) Post-surgery: 0 (0%)	Pre-surgery: 2 (40%) Post-surgery: 2 (40%)	Pre-surgery: 3 (11%) Post-Surgery: 3 (11%)	6 (24.0%)	58 (37.4%)	13 (68.4%)	23 (56.1%)	100 (41.7%)
Surgery duration, minutes (mean, SD)	72.7 (25.4)	394.8 (144.9)	623 (188.2)	222.3 (243.4)	N/A	N/C	N/C	N/C	N/C
Days before surgery (mean, SD, range)	7.7 (5.6, 1–22)	10.3 (4.8, 4–14)	7.2 (5.0, 1–14)	8 (5.3, 1–22)	N/A	N/A	N/A	N/A	N/A
Days since surgery (mean, SD, range)	13.2 (4.5, 5–21)	14.3 (3.7, 10–19)	15.6 (4.5, 10–21)	13.8 (4.4, 5–21)	N/A	27.7 (12.7, 2–56)	26.4 (13.1, 2–50)	32.7 (14.1, 10–59)	28.55 (13.09, 2–59)

Not all women in the RISE study had known or available nodal surgery type; N/A means not applicable; N/C means not collected.

#### Differences in HRQOL outcomes based on surgery type

**Fig. 1:**
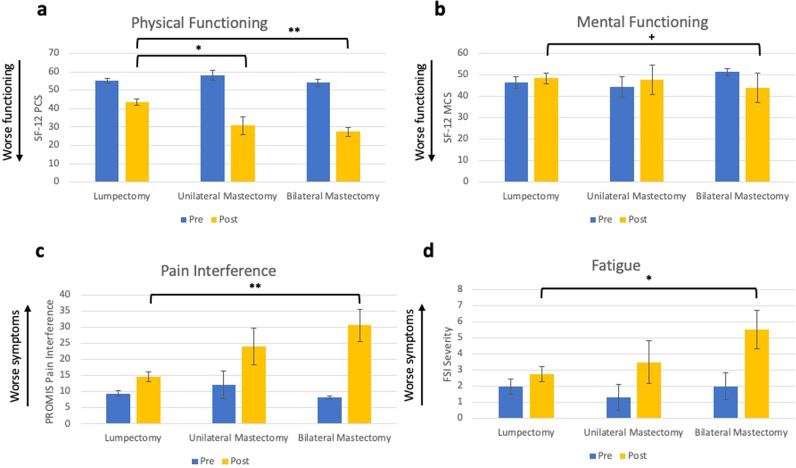
Study 1 pre- and post-surgical HRQOL outcomes. Average levels of physical functioning (**a**), mental functioning (**b**), pain interference (**c**), and fatigue (**d**) are presented with error bars representing standard errors. Differences between groups for post-surgical values are represented by bars with * indicating statistically significant differences using ANCOVA (* = *p* < 0.05, ** = *p* < 0.01) and ^+^ indicating a marginally significant difference (^+^ = *p* = 0.08).

Descriptive statistics of outcome variables and mean differences and *p* values for all post hoc tests for both studies are reported in Supplementary Tables 1 and 2, respectively. Surgery type accounted for significant differences (tested using analysis of covariance (ANCOVA)) in physical functioning (*F*(2,17) = 12.45, *p* < 0.001, Fig. 1a), pain interference (*F*(2,17) = 9.10, *p* = 0.002, Fig. 1c), and fatigue (*F*(2,17) = 4.15, *p* = 0.034, Fig. 1d), and was marginally associated with mental functioning (*F*(2,17) = 2.92, *p* = 0.08, Fig. 1b) and pain intensity (*F*(2,17) = 3.08, *p* = 0.072, data not shown). Post hoc analyses comparing lumpectomy and mastectomy revealed that unilateral mastectomy and bilateral mastectomy were associated with significantly poorer post-surgical physical functioning compared to lumpectomy (Cohen’s *d* for unilateral mastectomy = 1.49; Cohen’s *d* for bilateral mastectomy = 2.58). Further, bilateral mastectomy was associated with significantly worse pain interference (Cohen’s *d* = 1.73) and significantly greater fatigue than those who received lumpectomy (Cohen’s *d* = 1.17). Duration of the surgical procedure, which was greatest for bilateral mastectomy, was also significantly associated with changes in physical functioning, fatigue, and pain (bivariate associations are presented in Supplementary Table 4).

#### Differences in systemic inflammatory markers based on surgery type

**Fig. 2:**
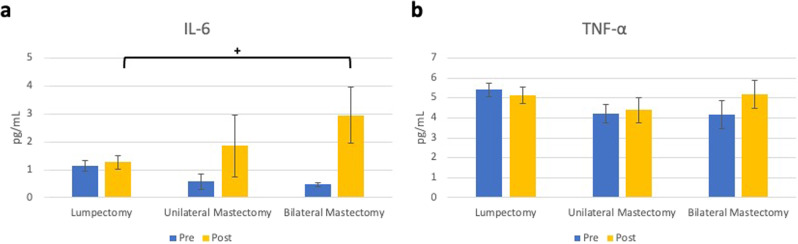
Study 1 pre- and post-surgical inflammatory markers. Average levels of IL-6 (**a**) and TNF-α (**b**) are presented with error bars representing standard errors. Marginally significant differences between groups for post-surgical values using ANCOVA are represented by bars. (^+^ = *p* = 0.056).

Surgery type accounted for marginally significant differences in post-surgical plasma IL-6 concentrations (tested using ANCOVA) (*F*(2,11) = 3.47, *p* = 0.068, Fig. 2a), but not TNF-α (*p* = 0.38, Fig. 2b). In post hoc analyses, bilateral mastectomy was associated with marginally significantly higher post-surgical IL-6 than lumpectomy (Cohen’s *d* = 1.23). Duration of surgery was also significantly associated with greater increases in log concentrations of both inflammatory markers (bivariate associations are presented in Supplementary Table 4).

#### Associations between systemic inflammatory markers and HRQOL outcomes

Increases in plasma IL-6 were associated with increases in pain interference (tested using multiple linear regression) (*b* = 7.85, SE = 3.71, *p* = 0.048) and fatigue (*b* = 2.63, SE = 0.86, *p* = 0.006), and with decreases in physical function (*b* = −12.04, SE = 4.64, *p* = 0.018). Increases in TNF-α were associated with increases in pain interference (*b* = 15.75, SE = 6.79, *p* = 0.032) and fatigue (*b* = 16.99, SE = 5.76, *p* = 0.008), but not with changes in physical functioning.

### Study 2

#### Patients

In all, 240 women met inclusion requirements for the current study and completed an enrollment questionnaire (Table 1). Of these, 25 were planning to have neoadjuvant chemotherapy and had not had a surgical procedure prior to enrollment. The enrollment visit, on average, took place 4 weeks following surgery for women who received one. Most women receiving mastectomy had immediate reconstruction (75%). Of the 240 women, 178 had blood available for inflammatory marker assays. Women who received bilateral mastectomies were on average younger than those receiving lumpectomy (tested using ANOVA) (*p* = 0.004). Additionally, the no-surgery group was younger than both lumpectomy and unilateral mastectomy groups (*ps* = 0.001 and 0.049, respectively).

#### Differences in HRQOL outcomes based on surgery type

**Fig. 3:**
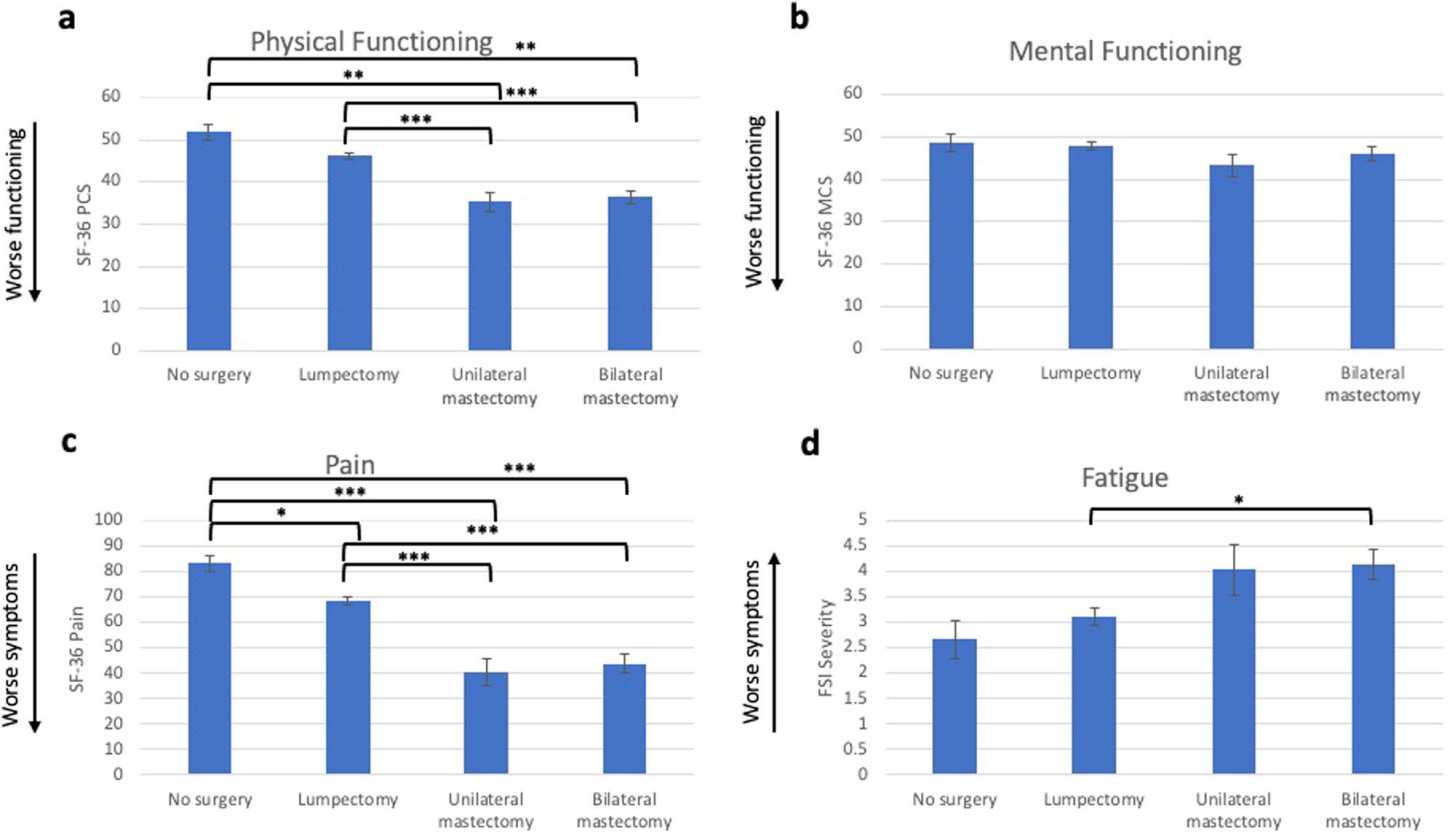
Study 2 HRQOL outcomes. Average levels of physical functioning (**a**), mental functioning (**b**), pain (**c**), and fatigue (**d**) are presented with error bars representing standard errors. Statistically significant differences between groups using ANCOVA are represented by bars (* = *p* < 0.05, ** = *p* < 0.01, *** = *p* < 0.001).

Surgery type accounted for significant variability in physical functioning (tested using ANCOVA) (*F*(3,222) = 19.83, *p* < 0.001, Fig. 3a), pain (F(3,222) = 24.98, *p* < 0.001, Fig. 3c), and fatigue (F(3,222) = 4.63, *p* = 0.004, Fig. 3d), but not mental functioning (Fig. 3b). Compared to the no-surgery group, women who received unilateral and bilateral mastectomies exhibited significantly lower physical functioning scores (Cohen’s *d* for unilateral mastectomy = 1.82; Cohen’s *d* for bilateral mastectomy = 1.67). When compared to the lumpectomy group, both unilateral mastectomy and bilateral mastectomy were also associated with poorer post-surgical physical functioning (Cohen’s *d* for unilateral mastectomy = 1.18; Cohen’s *d* for bilateral mastectomy = 1.04). All surgery groups exhibited worse scores on the SF-36 pain subscale than the no-surgery group (Cohen’s *d* for lumpectomy = 0.78; Cohen’s *d* for unilateral mastectomy = 2.18; Cohen’s *d* for bilateral mastectomy = 1.92). Again, both unilateral and bilateral mastectomy was associated with poorer outcomes than lumpectomy (Cohen’s *d* for unilateral mastectomy = 1.29; Cohen’s *d* for bilateral mastectomy = 1.09). For fatigue, only bilateral mastectomy differed from lumpectomy (Cohen’s *d* = 0.52).

#### Differences in systemic inflammatory markers based on surgery type

**Fig. 4:**
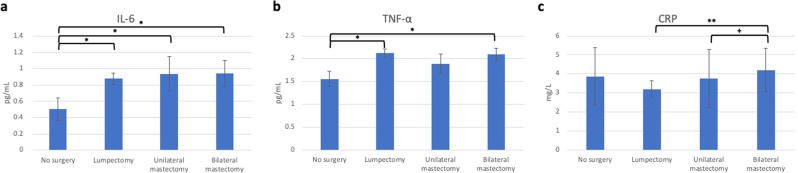
Study 2 inflammatory markers. Average levels of IL-6 (**a**), TNF-α (**b**), and CRP (**c**) are presented with error bars representing standard errors. Statistically significant differences between groups are represented by bars with * indicating statistically significant differences using ANCOVA (* = *p* < 0.05, ** = *p* < 0.01) and ^+^ indicating a marginally significant difference (^+^ = *p* = 0.052).

Surgery type accounted for significant variability in plasma IL-6 (tested using ANCOVA) (*F*(3,163) = 4.39, *p* = 0.005, Fig. 4a), TNF-α (*F*(3,163) = 3.30, *p* = 0.022, Fig. 4b), and CRP (*F*(3,163) = 4.08, *p* = 0.008, Fig. 4c). All three surgery groups exhibited significantly higher concentrations of IL-6 compared to the no-surgery group (Cohen’s *d* for lumpectomy = 0.87; Cohen’s *d* for unilateral mastectomy = 0.80; Cohen’s *d* for bilateral mastectomy = 0.81). There were no differences between the surgery groups. Women who received a lumpectomy or bilateral mastectomy had higher concentrations of TNF-α relative to the no-surgery group (Cohen’s *d’s* = 0.67 and 0.78, respectively). There were no differences between the surgery groups. Lastly, the bilateral mastectomy group exhibited significantly higher concentrations of CRP relative to the lumpectomy group (Cohen’s *d* = 0.28) and marginally higher concentrations relative to the unilateral mastectomy group (Cohen’s *d* = 0.08), but no different from the no-surgery group.

#### Associations between systemic inflammatory markers and HRQOL outcomes

Higher plasma IL-6 was associated with more pain (tested using multiple linear regression) (*b* = −6.92, SE = 2.65, *p* = 0.01) and fatigue (*b* = 0.61, SE = 0.22, *p* = 0.006), and with lower physical function (*b* = −3.19, SE = 1.10, *p* = 0.004). Higher TNF-α was marginally associated with more pain (*b* = −8.36, SE = 4.57, *p* = 0.069) and fatigue (*b* = 0.73, SE = 0.37, *p* = 0.05) and with lower physical function (*b* = −4.29, SE = 1.89, *p* = 0.024). Higher CRP was marginally associated with lower physical function (*b* = −1.29, SE = 0.66, *p* = 0.052).

## Discussion

In two studies of breast cancer patients, we found that extent of breast surgery was associated with both HRQOL and systemic inflammatory markers in the acute postoperative context and that higher concentrations of inflammatory markers were associated with poorer HRQOL. Across both studies, mastectomy (unilateral or bilateral) was associated with poorer physical functioning, and receipt of bilateral mastectomy was associated with greater pain and fatigue severity. Bilateral mastectomy in particular was associated with a larger than 2 standard deviation difference relative to lumpectomy on these outcomes. Interestingly, these results differ from those of historical breast cancer surgery studies which do not find consistent differences in HRQOL between breast conservation and mastectomy except for body image and sexual functioning^8^. These results are consistent; however, with the limited research on *contemporary* breast cancer surgical procedures which finds poorer physical functioning and more pain following mastectomy^14–16^. We also found that bilateral mastectomy was associated with higher levels of post-surgery systemic inflammatory markers relative to lumpectomy (Study 1) and that bilateral mastectomy was associated with higher levels of systemic inflammatory markers relative to both the no-surgery and lumpectomy groups at one month following surgery (Study 2). To our knowledge, this is the first study to compare differences in post-surgical systemic inflammatory biology following breast cancer surgery. Mental functioning did not significantly differ based on surgery type. Considering that women might opt for more extensive surgery for psychosocial reasons, our findings suggest that at least in the acute context, women with more extensive surgery do not exhibit better mental functioning.

There are several features of contemporary breast cancer surgery that might explain these differences. Mastectomy involves removing a substantially greater amount of tissue as well as a longer duration of surgery and anesthesia than does lumpectomy. In the SSS, unilateral mastectomies with immediate reconstruction on average took over 6.5 h, and bilateral over 10 h to complete whereas lumpectomies were performed in just over 1 h. Indeed, the duration of surgery was associated with changes in both HRQOL and inflammatory variables. Longer surgery times are driven in part by an immediate reconstruction that varies from autologous tissue transfer to expander with subsequent implantation. In the SSS, all mastectomy patients received immediate reconstruction and in RISE, most patients underwent immediate reconstruction. More extensive tissue injury might lead to higher levels of postoperative systemic inflammatory markers. For example, inflammatory mechanisms may be implicated in a higher likelihood of post-mastectomy pain syndrome, contributing to morbidity in these patients^21^. The extent to which post-surgical increased inflammation leads to greater post-adjuvant therapy persistent symptoms (i.e., following chemotherapy, radiation, or endocrine therapy) is an area in need of further investigation.

Results from these two studies are meant to provide enhanced information for clinicians and patients as they discuss the choice and impact of specific breast cancer surgical treatments, with or without reconstruction. This may be particularly important for minimizing treatment-associated morbidity in patients who are likely to be long-term cancer survivors. Understanding potentially modifiable variables that influence HRQOL in breast cancer survivorship is crucial given that poorer physical functioning is associated with higher mortality in this patient population^22,23^. Therefore, when mastectomy, and in particular bilateral mastectomy, is not clinically indicated, the surgical choice is a potentially modifiable variable for influencing the acute post-surgical impact on HRQOL. Patients should be informed about the acute side effects of more extensive surgery (i.e., mastectomy, especially when bilateral) when opting for more extensive surgery and that this will likely result in poorer post-operative HRQOL. It is possible that patients who have breast-conserving surgery and do not receive radiation therapy might avoid a radiation-induced inflammatory response. Previous research has shown transient effects of radiation on certain inflammatory markers (e.g., IL-6, sTNF)^24^, though not others (e.g., CRP)^25,26^, and effects of radiation may be magnified among patients who received both radiation and chemotherapy^27,28^. The acute and longer-term impact of different breast cancer therapies on markers of inflammation and measures of quality of life is an important question for future research. Of course, there are diverse reasons for choosing mastectomy among patients and their clinicians and patient preference may impact these patient-reported outcomes; our goal in reporting these findings is only to provide information about the impact of more extensive treatments, particularly as rates are increasing in the US.

Limitations to the current studies warrant consideration. First, the sample size of Study 1 was too small to interpret the significance. Although larger in total, Study 2 had relatively small sample sizes for the unilateral and bilateral mastectomy subgroups. Second, the RISE study no-surgery comparison group is comprised of significantly younger women preparing to have neoadjuvant chemotherapy (although age was controlled in all analyses). Thus, biological features of cancer may make these patients inherently different from those who had surgery first. Additionally, both samples had a relatively small number of Black and Hispanic patients and were primarily higher-income patients. Future research will need to examine these associations in more diverse samples with clinically matched no-surgery control groups. Additionally, missing data and differences between measures of pain and lack of CRP data for Study 1 limit comparability in findings between Studies 1 and 2. The acute findings here also do not account for the physical and financial burden stemming from the many additional procedures that patients with mastectomy and reconstruction require during the year following immediate reconstruction^29,30^. Future research is required to examine associations between contemporary breast cancer surgeries, HRQOL, and inflammation longer-term. Knowledge of the physical impact of mastectomies in the post-operative setting could provide enhanced information for patients regarding the burden of a more extensive surgery when making surgical choices for early-stage disease. These considerations are especially important when more extensive surgery is not clinically indicated, especially with the increased use of contralateral prophylactic mastectomy^31^, despite efforts to deimplement them^32,33^. These results may be particularly relevant to patients with DCIS where ongoing active surveillance in lieu of surgery may be an option for select women^34^.

## Methods

### Participants and procedures

#### Study 1: Surgical Symptoms Study (SSS)

##### Participants

SSS was designed to examine changes in pre- to post-surgery severity of physical, mental, and behavioral symptoms as well as inflammatory markers, in women with breast cancer. A primary goal was a comparison of the severity of post-treatment symptoms between surgery types. Patients were recruited from the UCLA Health breast cancer surgical practice. Women were eligible if they were aged 21 to 70 years and had been diagnosed with Stage 0-IIIA breast cancer. Enrollment began on 8/2013 and concluded on 10/2015. The research was approved by the University of California, Los Angeles, Institutional Review Board, and written informed consent was obtained from all participants.

##### Procedures

SSS participants were assessed ~1 week prior to breast surgery and again 1–3 weeks post-operatively. At both assessments, women completed surveys and had their blood drawn.

#### Study 2: Research on Inflammation, Stress, and Energy (RISE) Study

##### Participants

Participants for study 2 were drawn from the larger RISE study, which was designed to examine biobehavioral predictors of fatigue in women with breast cancer^35,36^. Patients were recruited from oncology practices in Los Angeles and were eligible if they had been diagnosed with Stage 0–IIIA breast cancer and had not yet received any adjuvant therapy. A total of 270 women were enrolled between 1/2013 and 7/2015. The majority of RISE participants were enrolled after surgery and before receipt of adjuvant therapy with radiation, chemotherapy, and/or endocrine therapy (*n* = 245, 91% of sample). Women scheduled to receive neoadjuvant chemotherapy were also included in RISE; however, they did not have surgery prior to enrollment (*n* = 25, 9% of sample). The institutional review boards at the University of California at Los Angeles and Cedars-Sinai Medical Center approved the study, and all participants provided written informed consent.

The current study focused on data from the baseline RISE visit, which was conducted before onset of adjuvant therapy (if indicated). We limited inclusion for this study to those who had received primary surgery within 60 days of enrollment in RISE and the baseline visit (*n* = 215, 30 women excluded). We also included women who were scheduled to receive neoadjuvant chemotherapy and had not had surgery prior to enrollment, who served as the no-surgery comparison group (*n* = 25).

##### Procedures

At the pre-adjuvant therapy enrollment assessment, women completed surveys and had their blood drawn.

### Measures

Demographic characteristics were obtained from self-reports at enrollment in both studies and included age, race/ethnicity, income, education, and employment status. Disease and treatment-related information was obtained from medical record abstraction and included cancer stage, type of surgery received, length of surgery (for SSS only), and time since surgery.

HRQOL outcomes including physical and mental functioning, pain, and fatigue were assessed using standard questionnaires. SSS participants completed the 12-item Short Form Health Survey (SF-12) from which the Physical and Mental Component Scales (PCS and MCS) were derived (higher scores indicate better functioning)^37^. Both measures have population means of 50 points and standard deviations of 10 points. RISE participants completed the SF-36 which was also used to derive the PCS and MCS^38^. For pain, the SSS included the PROMIS pain interference and pain intensity scales (higher scores indicate greater pain)^39,40^, and the RISE study used the SF-36 pain subscale (higher scores indicate less pain)^38^. For fatigue, both studies administered the Fatigue Symptom Inventory (FSI) severity scale (higher scores indicate greater fatigue)^41^.

Systemic inflammatory markers were assessed by quantifying concentrations of plasma interleukin (IL)−6, tumor necrosis factor-alpha (TNF-α), and C-reactive protein (CRP, RISE only). These inflammatory markers are commonly elevated in the context of breast cancer treatments and survivorship and linked with behavioral symptoms such as fatigue and pain^42–44^. Blood samples were collected by venipuncture, processed for plasma, and stored at – 80 °C until assayed at the UCLA Cousins Center for Psychoneuroimmunology. IL-6 and TNF-α concentrations were measured in a multiplex assay utilizing a V-PLEX Custom Human Cytokine Proinflammatory Panel on the Meso Scale Discovery (MSD) electrochemiluminescence platform and Discovery Workbench software (MSD, Rockville, MD). CRP was measured by Human Quantikine ELISA (R&D Systems, Minneapolis, MN) according to the manufacturer’s protocols with a lower limit of 0.2 mg/L. Two samples had CRP concentrations above the range of the standard curve (25 mg/L) and were estimated using extrapolated values. All samples were assayed in duplicate and averaged for analysis. For all plasma biomarkers, inter-assay coefficients of variation were less than or equal to 10%, and mean intra-assay coefficients of variation were <5%. To correct for skewness, all analyses used log-transformed inflammatory marker values, and figures and results provide unadjusted values for interpretability.

### Statistical analyses

#### Study 1: SSS

ANCOVA was used to test the differences between surgery types in post-surgical HRQOL and inflammatory markers while controlling for pre-surgery values and age, stage, and days since surgery. BMI and education were included as covariates for inflammatory marker analyses only given associations between BMI and socioeconomic status on circulating inflammatory markers^45,46^. Post hoc pairwise comparisons with Tukey adjustments for multiple comparisons were used to test differences between surgery types (lumpectomy, unilateral mastectomy, bilateral mastectomy) on post-surgery values. Effect sizes were computed using Cohen’s *d* and based on post-surgery values to facilitate comparisons with Study 2. Unadjusted values of inflammatory markers were employed for effect sizes. Multiple linear regression analyses were conducted to examine associations between changes in inflammatory markers and significant changes in HRQOL outcomes from pre- to post-surgery, controlling for age and BMI. All tests of significance were two-sided.

#### Study 2: RISE

ANCOVA was used to test the differences between surgery types in baseline HRQOL and inflammatory markers while controlling for age, stage, and days since surgery. BMI and education were included as covariates for inflammatory marker analyses only given associations between BMI and socioeconomic status on circulating inflammatory markers^45,46^. Post hoc pairwise comparisons with Tukey adjustments for multiple comparisons were used to test differences between surgery types (no-surgery, lumpectomy, unilateral mastectomy, bilateral mastectomy). Effect sizes were computed using Cohen’s *d* and unadjusted values of inflammatory markers. Multiple linear regression analyses were conducted to examine associations between inflammatory markers and significant HRQOL outcomes controlling for age and BMI. All tests of significance were two-sided.

All analyses were conducted using Stata software, version 16.1 for Mac.

## Supplementary information

Supplementary Information (pdf)

## Data Availability

The data sets generated and analyzed during the current study are available from the corresponding author on reasonable request.

## Code availability

The codes generated during the current study are available from the corresponding author on reasonable request.
