# Supplementary material for: Acute health-related quality of life outcomes and systemic inflammatory markers following contemporary breast cancer surgery
Source: NPJ Breast Cancer. 2022 Aug 8;8:91. doi: 10.1038/s41523-022-00456-4 (PMC9359976; doi:10.1038/s41523-022-00456-4)
Supplement: Supplementary file 1 — Supplementary Information (pdf) [file 41523_2022_456_MOESM1_ESM.pdf]

Supplementary Table 1: Descriptive statistics for significant outcome variables

	Study 1 (Surgical Symptoms Study)						Study 2 (RISE Study)		
	Pre-Surgery Assessment			Post-Surgery Assessment			Enrollment (Post-Surgery) Assessment		
	Mean	SD	Range	Mean	SD	Range	Mean	SD	Range
Physical Functioning (MOS Short Form Health Survey)									
• Lumpectomy	55.20	6.02	40.69-65.28	43.61	7.06	30.12-56.58	46.16	9.42	23.45-61.31
• Unilateral Mastectomy	58.00	5.39	50.76-63.72	30.94	9.75	20.63-42.27	35.26	9.10	22.48-56.01
• Bilateral Mastectomy	54.13	4.40	49.19-60.28	27.49	5.34	20.95-34.28	36.41	9.31	23.18-59.41
• No Surgery							51.84	9.15	24.75-60.13
Pain (Study 1: PROMIS pain interference; Study 2: MOS Short Form Health Survey)									
• Lumpectomy	9.50	3.31	8-20	14.67	6.97	8-36	68.72	21.25	23-100
• Unilateral Mastectomy	12.25	8.50	8-25	24.00	11.43	14-40	40.37	22.86	0-90
• Bilateral Mastectomy	8.40	0.89	8-10	30.6	11.04	12-40	43.85	24.35	0-100
• No Surgery							83.52	16.23	45-100
Fatigue (Fatigue Symptom Inventory severity)									
• Lumpectomy	1.98	1.95	0-6.33	2.78	2.01	0-6.67	3.12	2.08	0-8
• Unilateral Mastectomy	1.33	1.63	0-3.33	3.50	2.65	1.33-7.33	4.05	2.17	1-7
• Bilateral Mastectomy	2.00	1.84	0-5	5.53	2.67	2.33-8.00	4.15	1.85	0-8
• No Surgery							2.68	1.86	0-6
IL-6 (pg/mL)									

	Study 1 (Surgical Symptoms Study)						Study 2 (RISE Study)		
	Pre-Surgery Assessment			Post-Surgery Assessment			Enrollment (Post-Surgery) Assessment		
	Mean	SD	Range	Mean	SD	Range	Mean	SD	Range
• Lumpectomy	1.13	0.85	0.30-2.90	1.27	0.92	0.30-3.90	0.88	0.76	0.10-5.10
• Unilateral Mastectomy	0.58	0.55	0.10-1.30	1.85	2.24	0.60-5.20	0.94	0.77	0.10-2.40
• Bilateral Mastectomy	0.48	0.15	0.30-0.60	2.95	1.99	1.30-5.30	0.94	0.89	0.15-4.30
• No Surgery							0.50	0.61	0.10-2.80
TNF-a (pg/mL)									
• Lumpectomy	5.42	1.46	2.90-7.40	5.15	1.57	2.90-8.20	2.12	0.95	0.60-7.40
• Unilateral Mastectomy	4.23	0.91	2.90-5.00	4.40	1.25	2.60-5.30	1.89	0.76	0.40-3.30
• Bilateral Mastectomy	4.18	1.39	3.00-5.90	5.18	1.41	3.60-7.00	2.09	0.73	0.70-3.80
• No Surgery							1.56	0.71	0.70-3.10
CRP (pg/L)									
• Lumpectomy							3.19	4.69	0.10-23.50
• Unilateral Mastectomy							3.75	5.50	0.10-16.90
• Bilateral Mastectomy							4.20	6.35	0.30-31.00
• No Surgery							3.87	6.58	0.30-29.00

Supplementary Table 2: Mean differences and inferential statistics for statistically or marginally significant post-hoc comparisons

	Study 1 (Surgical Symptoms Study)			Study 2 (RISE Study)		
	Mean difference	p	Cohen's d	Mean difference	p	Cohen's d
Physical Functioning						
• Lumpectomy vs. Unilateral Mastectomy	12.67	.022	1.49	10.90	<.001	1.18
• Lumpectomy vs. Bilateral Mastectomy	16.12	.001	2.58	9.75	<.001	1.04
• No surgery vs. Unilateral Mastectomy				16.58	.012	1.82
• No surgery vs. Bilateral Mastectomy				15.44	.013	1.67
Pain						
• Lumpectomy vs. Unilateral Mastectomy				28.35	<.001	1.29
• Lumpectomy vs. Bilateral Mastectomy	15.93	.002	1.73	24.87	<.001	1.09
• No surgery vs. Lumpectomy				14.79	.007	.078
• No surgery vs. Unilateral Mastectomy				43.15	<.001	2.18
• No surgery vs. Bilateral Mastectomy				39.67	<.001	1.92
Fatigue						
• Lumpectomy vs. Bilateral Mastectomy	2.76	.029	1.17	1.02	.017	.52
IL-6 (pg/mL)						
• Lumpectomy vs. Bilateral Mastectomy	1.68	.056	1.23			
• No surgery vs. Lumpectomy				0.37	.013	0.87
• No surgery vs. Unilateral Mastectomy				0.43	.010	0.80
• No surgery vs. Bilateral Mastectomy				0.44	.006	0.81

	Study 1 (Surgical Symptoms Study)			Study 2 (RISE Study)		
	Mean difference	p	Cohen's d	Mean difference	p	Cohen's d
TNF-a (pg/mL)						
• No surgery vs. Lumpectomy				0.56	.044	
• No surgery vs. Bilateral Mastectomy				0.54	.022	0.78
CRP (pg/L)						
• Lumpectomy vs. Bilateral Mastectomy				1.01	.005	0.28

Supplementary Table 3: Descriptive statistics for outcomes based on reconstruction type

	Study 1 (Surgical Symptoms Study)					
	Pre-Surgery Assessment			Post-Surgery Assessment		
	Mean	SD	Range	Mean	SD	Range
Physical Functioning (MOS Short Form Health Survey)						
• Autologous	54.36	5.15	49.19-60.28	27.24	7.16	20.63-35.42
• Implant-based	57.71	4.74	52.20-63.72	31.24	7.86	25.42-42.27
Pain (PROMIS pain interference)						
• Autologous	11.80	7.43	8-25	26.00	12.41	12-40
• Implant-based	8.00	0	8-8	29.75	10.47	18-40
Fatigue (Fatigue Symptom Inventory severity)						
• Autologous	1.67	1.20	0-3.33	4.73	2.70	2.33-8
• Implant-based	1.75	2.36	0-5	4.50	3.13	1.33-7.67
IL-6 (pg/mL)						
• Autologous	0.32	0.19	0.10-0.60	3.34	2.08	1.00-5.30
• Implant-based	0.87	0.38	0.60-1.30	0.83	0.40	0.60-1.30
TNF-a (pg/mL)						
• Autologous	4.64	1.01	3.10-5.90	5.28	1.20	3.60-7.00
• Implant-based	3.47	0.90	2.90-4.50	3.97	1.19	2.60-4.80

Supplementary Table 4: Study 1 bivariate associations between surgery length and changes in HRQOL and inflammatory markers

	r	*p*
Physical Functioning (MOS Short Form Health Survey)	-0.62	< .001
Pain (PROMIS pain interference)	0.60	< .001
Fatigue (Fatigue Symptom Inventory severity)	0.60	< .01
IL-6 (pg/mL)	0.72	< .001
TNF-a (pg/mL)	0.41	0.049
